# Two parameters reflect lipid-driven inflammatory state in acute coronary syndrome: atherogenic index of plasma, neutrophil–lymphocyte ratio

**DOI:** 10.1186/s12872-016-0274-7

**Published:** 2016-05-17

**Authors:** Youqin Zhan, Tan Xu, Xuerui Tan

**Affiliations:** Department of Cardiology, First Affiliated Hospital of Shantou University Medical College, Shantou, Guangdong 515041 China

**Keywords:** Acute coronary syndrome, Inflammation, Lipid profile, Atherogenic index of plasma, Neutrophil–lymphocyte ratio

## Abstract

**Background:**

Atherosclerosis is a systemic, lipid-driven immune-inflammatory disease.

**Methods:**

We retrospectively reviewed institutional electronic medical records to seek chest pain patients who were suspicious of acute coronary syndrome (ACS) between January 2011 and December 2013. All the patients were identified by undergoing coronary angiography. On admission white blood cell and its subtypes were measured as part of the automated complete blood count and fasting venous blood samples were obtained and analyzed for lipids profiles used automated analysis.

**Results:**

A total of 376 consecutive patients with ACS were investigated. In the same period, 378 patients admitted with chest pain suspicious of ACS were also included in this study for control. Blood glucose, serum creatinine, white blood cell, neutrophil and monocyte were insignificantly higher in the ACS group. ACS group had higher total cholesterol and lower high density lipid-cholesterol. However, triglyceride and low density lipid-cholesterol were similar between ACS and control groups. Atherogenic index of plasma (AIP) was significantly higher in ACS group compared to control group (*p* = 0.029). Similarly, ACS group had higher neutrophil–lymphocyte ratio (NLR) than those in control group. In the subgroups, the NLR were significantly higher in the STEMI group (*p* < 0.001). However, AIP were similar between the three subgroups (*p* = 0.748).

**Conclusions:**

Our data firstly investigated the lipid-driven inflammatory state in acute coronary syndrome through two easily feasible parameters. There suggest that there are higher AIP and NLR in the ACS patients. Moreover, ACS subgroups are all lipid-driven states, but inflammation levels are different in the entity ACS subgroups.

## Background

Coronary heart disease (CHD) is leading cause of morbidity and mortality worldwide [1]. CHD is mainly caused by coronary atherosclerosis with or without luminal thrombosis and vasospasm [2]. Atherosclerosis is a systemic, lipid-driven immune-inflammatory disease [3]. The function of inflammation in the development and progression of atherosclerosis has been clarified [4]. The presence of inflammation at the site of the atherosclerotic lesion has a critical pathophysiological role in acute rupture [5].

In recent years, several studies have been demonstrated that White blood cell (WBC) count and its subtypes are also known as classic markers of inflammation in cardiovascular diseases [6–9]. Palmerini T showed that a high neutrophil–lymphocyte ratio (NLR) is a strong and independent predictor of in-hospital cardiovascular mortality due to ST-segment elevation myocardial infarction (STEMI) [6].

Dyslipidemia is a well-established risk factor for the development of atherosclerosis which is lipid-driven disease [10, 11]. The common combined occurrence of high fasting blood concentrations of triglycerides (TG) and low levels of high density lipoprotein cholesterol (HDL-c) has been referred to as atherogenic dyslipidemia [12]. Dobiasova and Frohlich have referred to this log_10_ [TG/HDL-c] as the atherogenic index of plasma (AIP) that allows the demonstration of a correlation with smaller low-density lipoprotein- cholesterol (LDL-c) particles and increased fractional esterification rate for cholesterol in plasma [13, 14]. Interestingly, AIP is associated more strongly with elevated C-reactive protein levels [12]. However, fewer studies evaluate AIP and classic markers of inflammation together. There may be links between NLR and AIP in patients with acute coronary syndrome (ACS) according to the pathophysiologic of atherosclerosis, lipid-driven immune-inflammatory disease [3].

We, therefore, aimed to determine the relationships between classic markers of inflammation (white blood cell and its subtypes) and AIP in acute coronary syndrome, also to evaluate the value the aforementioned parameters in the differential diagnoses of chest pain patients.

## Methods

### Study design and definition

We retrospectively reviewed institutional electronic medical records to seek chest pain patients who were suspicious of acute coronary syndrome between January 2011 and December 2013. All the patients were identified by undergoing coronary angiography. ACS group included STEMI, non-ST elevated myocardial infarction (NSTEMI) and unstable angina (UA) that were defined based on the criteria formulated by updated guidelines [15, 16]. Patients in the control group were certified the coronary artery normal by angiography. The chief complaint of all the included patients was chest pain. All the patients’ medical records were reviewed to extract the demographic and clinical data by two physicians (Youqin Zhan and Tan Xu).

Hypertension was determined if the systolic BP (SBP) was ≥140 mm Hg and/or the diastolic BP (DBP) was over 90 mm Hg and/or use of antihypertensive medication [17]. Individuals with type 2 diabetes were diagnosed according to the criteria of the American Diabetes Association, [18] namely when plasma fasting glucose was more than 7.0 mmol/L (or 2-h postprandial glucose ≥11.1 mmol/L) and/or if there was current use of diabetes medication.

Patients with clinical evidence of active infection, cancer, hematological disease, systemic inflammatory conditions, autoimmune disease, liver disease, and renal failure were excluded from the study. As well as, patients with a history of prior myocardial infarction (MI), cerebrovascular event, peripheral arterial disease, percutaneous coronary intervention, coronary bypass surgery, and treatment with anti-platelet or statins were also excluded.

### Laboratory analysis

On admission WBC and its subtypes were measured as part of the automated complete blood count (Beckman counter LH780, USA). Fasting venous blood samples were obtained and analyzed for lipids, liver and kidney function used automated analysis (Beckman counter AU5800, USA). The NLR and AIP was calculated by EXCEL (Microsoft Excel). The NLR was calculated as the ratio of the neutrophils and lymphocytes, both obtained from the same automated blood sample at admission. AIP was defined as logarithm of this ratio (TG/HDL-C]) on the base of 10, which corrects for the lack of normative distribution of the ratio [14].

### Statistical analysis

The data were stored using the MS Excel program. The statistical analysis was carried out using SPSS for Windows release 13.0. Continuous variables are expressed as means and SD, as counts and percentages for Categorical variables. The conformity of all parameters to a normal distribution was tested using the Kolmogorov-Smirnov test. The independent *t* test and oneway ANOVA test were used for continuous variables. The *x*^2^ test was performed for the comparison of categorical variables. A value of *p* < .05 on the two-sided test was considered statistically significant.

This study was submitted to and approved by the Research and Ethics Committee of First Affiliated Hospital of Shantou University Medical College.

## Results

### Baseline characteristics

In this case-control retrospective study, a total of 376 consecutive patients with ACS were investigated. In the same period, 378 patients admitted with chest pain suspicious of ACS were also included in this study for control. Comparison of clinical and biochemical variables in the ACS and control groups are shown in Table 1. All the Subjects hadn’t the history of previous use of statin. The patients in the ACS group were older than those in the control group. Body mass index, rate of smoking, history of hypertension were not different between the ACS and control groups. Blood glucose, serum creatinine, white blood cell, neutrophil and monocyte were insignificantly higher in the ACS group. On lipid profiles, ACS group had higher total cholesterol and lower HDL-c. However, TG and LDL-c were similar between ACS and control groups.

**Table 1:** Comparison of clinical and biochemical variables in the ACS and control groups

	ACS group (*n* = 376)	Control group (*n* = 378)	*p* value
Age (years)	63.60 ± 11.74	59.81 ± 9.47	<0.001^b^
Male gender (%)	206 (54.79)	199 (52.65)	0.560^a^
BMI (kg/m^2^)	23.51 ± 2.08	22.76 ± 1.18	0.510^b^
Smoker (%)	250 (66.49)	174 (46.03)	0.070^a^
History of hypertension (%)	168 (44.68)	184 (48.68)	0.275^a^
History of diabetes mellitus (%)	84 (22.34)	46 (12.17)	<0.001^a^
SBP (mmHg)	130.99 ± 24.26	136.54 ± 20.48	0.001^b^
DBP (mmHg)	80.60 ± 14.50	82.54 ± 12.66	0.050^b^
Blood glucose (mmol/L)	9.23 ± 11.03	5.96 ± 1.84	<0.001^b^
Serum creatinine (ummol/L)	101.84 ± 38.57	88.17 ± 29.99	<0.001^b^
WBC (*10^9^/L)	10.83 ± 3.65	7.60 ± 2.49	<0.001^b^
Neutrophil (*10^9^/L)	7.98 ± 3.44	5.33 ± 10.25	<0.001^b^
Lymphocyte (*10^9^/L)	1.93 ± 0.92	2.08 ± 0.74	0.016^b^
Monocyte (*10^9^/L)	0.68 ± 0.33	0.513 ± 0.213	<0.001^b^
Hemoglobin (g/L)	138.62 ± 66.94	134.27 ± 14.21	0.217^b^
TC (mmol/L)	5.09 ± 1.30	4.86 ± 1.20	0.015^b^
TG (mmol/L)	1.62 ± 1.15	1.83 ± 5.13	0.402^b^
HDL-c (mmol/L)	1.11 ± 0.34	1.24 ± 0.62	<0.001^b^
LDL-c (mmol/L)	3.31 ± 1.09	3.67 ± 7.37	0.367^b^

*BMI* body mass index, *SBP* systolic blood pressure, *DBP* diastolic blood pressure, *WBC* white blood cell, *TC* total cholesterol, *TG* triglyceride, *HDL-c* high density lipid-cholesterol, *LDL-c* low density lipid-cholesterol. ^a^Chi-square test. ^b^Independent *t* test

### Lipid-driven inflammatory state-AIP and NLR

**Fig. 1:**
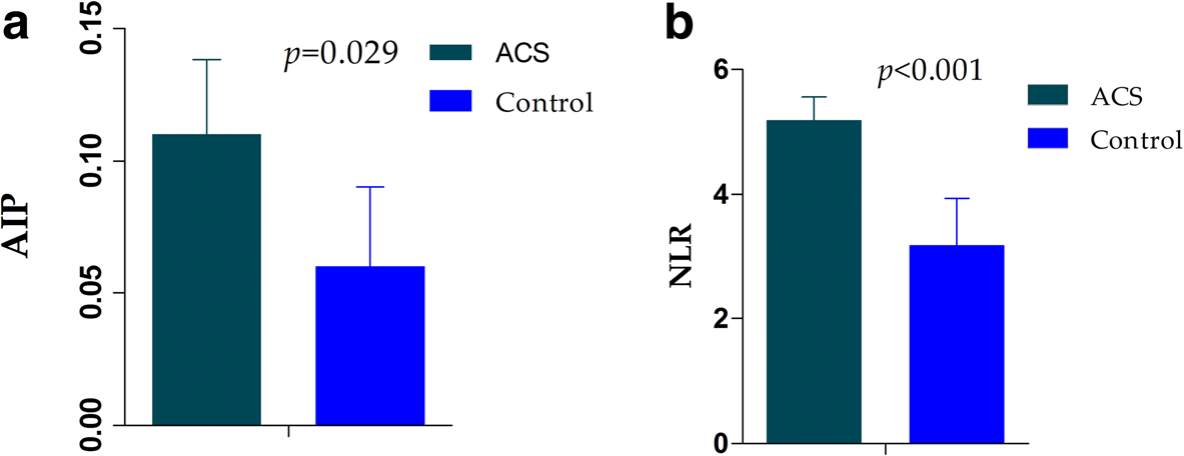
Comparison AIP (1**a**), NLR (1**b**) between the ACS and control groups

All the included patients were suspicious of acute coronary syndrome: lipid-driven inflammatory state. Two parameters, AIP and NLR, maybe reflect the state. As was to be expected, AIP was significantly higher in ACS group compared to control group (*p* = 0.029, shown in Fig. 1a). Similarly, ACS group had higher NLR than those in control group (*p* < 0.001, shown in Fig. 1b).

**Fig. 2:**
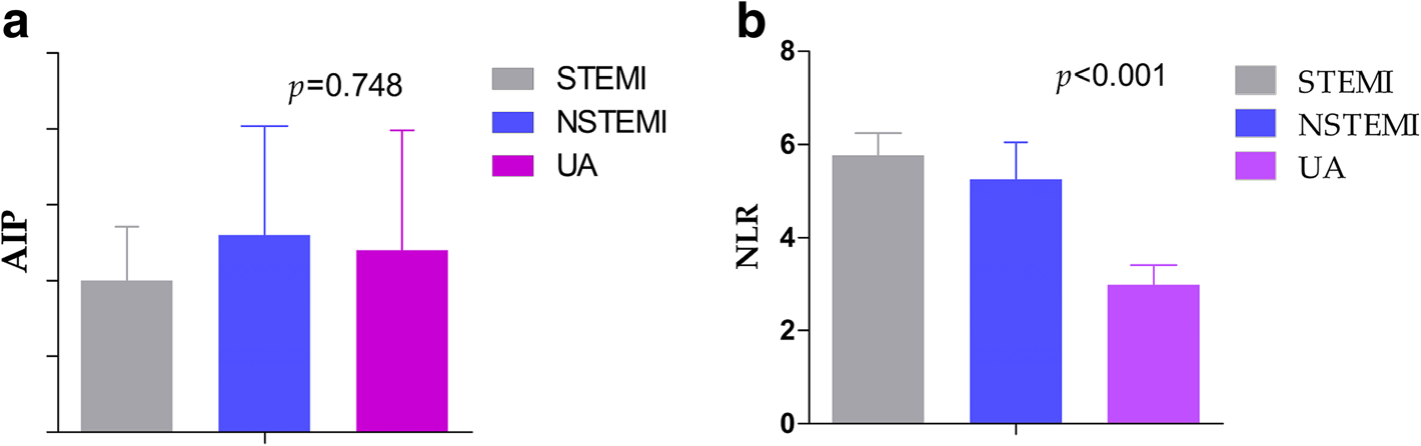
Comparison AIP (2**a**), NLR (2**b**) between the ACS subgroups

In order to further analyze the lipid-driven inflammatory state in ACS, the ACS groups were divided into three subgroups, namely, STEMI, NSTEMI and UA groups. Of the 376 ACS patients included, 237 (63.03 %) had STEMI, 74 (19.68 %) had NSTEMI and 65 (17.29 %) had UA. Table 2 shows the baseline characteristic of the aforementioned three groups. Mean ages, male genders and body mass index were similar in the three groups (*p* = 0.234, *p* = 0.078 and *p* = 0.172, respectively). Smoking was more frequently in the STEMI group (*p* = 0.033). Rate of hypertension was higher in the UA groups (*p* = 0.002). However, the frequencies of diabetes mellitus were similar between the three groups (*p* = 0.194). On the inflammation profiles, white blood cell, neutrophil and monocyte were lower in the UA groups than the other two groups (*p* < 0.001, *p* < 0.001 and *p* = 0.004, respectively). On the lipid profiles, there were no differences in total cholesterol, triglyceride, high density lipid-cholesterol and low density lipid-cholesterol between the three subgroups (*p* = 0.189, *p* = 0.578, *p* = 0.702, and *p* = 0.574, respectively). In the subgroups, the NLR were significantly higher in the STEMI group (*p* < 0.001, shown in Fig. 2a). However, AIP were similar between the three subgroups (*p* = 0.748, shown in Fig. 2b).

**Table 2:** The baseline characteristic of STEMI, NSTEM and UA groups

	STEMI group *n* = 237	NSTEMI group *n* = 74	UA group *n* = 65	*p* value
Age (years)	62.81 ± 11.45	64.91 ± 13.05	64.98 ± 11.11	0.234^b^
Male gender (%)	129 (54.43)	39 (52.70)	38 (58.46)	0.078^a^
BMI (kg/m^2^)	23.66 ± 1.99	23.16 ± 2.28	23.38 ± 2.22	0.172^b^
Smoker (%)	169 (71.31)	42 (56.76)	39 (60.00)	0.033^a^
History of hypertension (%)	90 (37.97)	39 (52.70)	39 (60.00)	0.002^a^
History of diabetes mellitus (%)	48 (20.25)	16 (21.62)	20 (30.77)	0.194^a^
SBP (mmHg)	127.07 ± 21.54	134.45 ± 30.66	141.34 ± 22.07	<0.001^b^
DBP (mmHg)	79.00 ± 13.49	82.42 ± 17.48	84.37 ± 13.53	0.014^b^
Blood glucose (mmol/L)	9.94 ± 13.60	8.55 ± 3.33	7.40 ± 3.57	0.218^b^
Serum creatinine (ummol/L)	101.50 ± 37.56	104.78 ± 43.93	99.75 ± 36.06	0.727^b^
WBC (*10^9^/L)	11.45 ± 3.56	11.14 ± 3.82	8.16 ± 2.43	<0.001^b^
Neutrophil (*10^9^/L)	8.63 ± 3.35	8.18 ± 3.60	5.32 ± 2.15	<0.001^b^
Lymphocyte (*10^9^/L)	1.88 ± 0.94	1.98 ± 0.94	2.07 ± 0.81	0.302^b^
Monocyte (*10^9^/L)	0.70 ± 0.34	0.74 ± 0.38	0.56 ± 0.24	0.004^b^
Hemoglobin (g/L)	136.67 ± 16.70	150.49 ± 147.18	132.13 ± 15.24	0.210^b^
TC (mmol/L)	5.14 ± 1.29	5.08 ± 1.27	4.92 ± 1.35	0.189^b^
TG (mmol/L)	1.59 ± 1.12	1.75 ± 1.39	1.59 ± 0.94	0.578^b^
HDL-c (mmol/L)	1.11 ± 0.31	1.14 ± 0.47	1.10 ± 0.27	0.702^b^
LDL-c (mmol/L)	3.36 ± 1.07	3.24 ± 1.14	3.23 ± 1.08	0.574^b^

*BMI* body mass index, *SBP* systolic blood pressure, *DBP* diastolic blood pressure, *WBC* white blood cell, *TC* total cholesterol, *TG* triglyceride, *HDL-c* high density lipid-cholesterol, *LDL-c* low density lipid-cholesterol. ^a^Chi-square test. ^b^oneway ANOVA test

## Discussion

Our data firstly investigated the lipid-driven inflammatory state in acute coronary syndrome through two easily feasible parameters. Retrospective evaluation of the AIP and NLR as a lipid and inflammation profiles in ACS patients yielded the following salient information. The main conclusions were that: (i) there were higher AIP and NLR in the ACS patients compared with other chest pain patients suspicious of ACS; and (ii) In the ACS subgroups, the NLR were significantly higher in the STEMI group, however, AIP were similar.

Regarding lipids profiles in ACS patients, there has been accumulated several clinical trials to demonstrate the potential role of lipid in the development of ACS. A statistically significant association between thrombosis caused by plaque rupture and high TC, low high-density lipoprotein cholesterol, and high TC/HDL-C ratio in men has been evaluated by Burke et al. [19]. In this study, there found that significantly higher TC and lower HDL-c in ACS group.

Recently, more studies have been focused on the parameter-AIP, which is a surrogate of small LDL particle size. Altan O. et al found that high AIP is a strong risk factor for CAD in both Turkish men and women [12]. Similarly, in the present study, AIP also was also higher in the ACS groups compared with control groups. However, between the subgroups of ACS, there were no difference in TC, HDL-c and AIP, which may reflects the coherence of ACS subgroups. Otherwise, a recent study [20] showed that the TC/HDL ratio was a marker of severity of CAD in relation to the number of vessels affected in patients with ACS without ST-segment elevation.

On the inflammation of ACS, it has been shown to play a role in the pathogenesis of coronary atherosclerosis [21]. In recent years, higher white blood cell [22] (WBC), and neutrophil [23, 24], have been evaluated to be associated with ACS. It has been shown that elevated baseline WBC count was associated with higher short-term mortality in UA and NSTEMI [25]. A recent study [22] presented that WBC was strongly associated with CHD. In terms of neutrophil, more studies described that elevated neutrophil may predict worse outcomes, such as chronic heart failure, [24] cardiogenic death [23]. Our study shows that a significantly higher WBC and neutrophil in the ACS group, and there also exists significantly differences in WBC and neutrophil between the ACS subgroups.

Moreover, NLR was introduced as a potential marker to determine inflammation in cardiac disorder and shown as a predictor of long-term mortality in ACS patients [26, 27]. Ramazan C. O. et al reported that NLR was significantly associated with adverse in-hospital outcomes, independent of GRACE risk score in STEMI patients. In the present study, ACS patients also had higher NLR compared to angiographic normal patients. Moreover, the NLR had significant differences in the ACS subgroups, highest in the STEMI subgroup.

The most challenging finding of the present study was to demonstrate the lipid-driven inflammation state in ACS patients used two combination parameters- AIP and NLR. To the best of our knowledge, no studies have assessed the potential role of AIP and NLR together in ACS patients to evaluate the lipid-driven inflammation state. Unlikely many other marker and bioassays, AIP and NLR are inexpensive and readily available markers that provides an additional level of risk scores in predicting short and long term outcomes in ACS patients. In this study, there are differences of AIP and NLR between ACS and control groups. However, in the ACS subgroups, although no difference found in AIP, yet there were differences in NLR. In the view of above differences, there may interpret as the following in leading pathological terms. Firstly, ACS is a systemic, lipid-driven inflammatory disease. Therefore, compared to angiographic normal patients, ACS groups had higher AIP and NLR, which reflect the lipid and inflammation status. Secondly, in the ACS groups, there are all lipid-driven states, so there is no difference in AIP. However, there may be different inflammation levels in the ACS groups. Lastly, coincidence may exist in the differences.

### Study limitations

The present study must be interpreted within the context of its potential limitations. First, this was a retrospective and single-center pilot study. Further prospective multi-center studies with a larger sample size may be needed. Second, the patients in NSTEMI and UA subgroups were smaller compared with the STEMI subgroups which may disturb the results. Third, we could not compare AIP and NLR with other lipid and inflammatory markers, because they were not routinely assessed in our study population.

## Conclusion

Our data suggest that there are higher AIP and NLR in the ACS patients, which evaluate the lipid-driven inflammation state, used two inexpensive parameters. Moreover, ACS subgroups are all lipid-driven states, but inflammation levels are different in the entity ACS subgroups. One challenge of the study is that we firstly evaluate the lipid-driven inflammation state of ACS used two ratio parameters together, AIP and NLR. Further studies are required to confirm the clinical relevance of these findings by comparing the association of AIP and NLR together with clinical outcomes.

### Ethics approval and consent to participate

This study was submitted to and approved by the Research and Ethics Committee of First Affiliated Hospital of Shantou University Medical College. This is a case-control retrospective study, so the content to participate is not applicable.

### Consent for publication

Not applicable.

### Availability of data and materials

The datasets supporting all the conclusions of this article are included within the article.

## Abbreviations

CHD: coronary heart disease
ACS: acute coronary syndrome
STEMI: ST-segment elevation myocardial infarction
NSTEMI: non-ST elevated myocardial infarction
UA: unstable angina
MI: myocardial infarction
WBC: white blood cell
NLR: neutrophil–lymphocyte ratio
TG: triglycerides
HDL-c: high density lipoprotein cholesterol
AIP: atherogenic index of plasma
LDL-c: low-density lipoprotein- cholesterol
